# Supplementary material for: The interactome of intact mitochondria by cross-linking mass spectrometry provides evidence for coexisting respiratory supercomplexes
Source: Mol Cell Proteomics. 2017 Dec 8;17(2):216–32. doi: 10.1074/mcp.RA117.000470 (PMC5795388; doi:10.1074/mcp.RA117.000470)
Supplement: Supplemental Data [file supp_RA117.000470_133922_0_supp_23978_fzffwf.zip › spectra_annotation/mito_spectra_annotation/46-1-10-1-19-1.pdf]

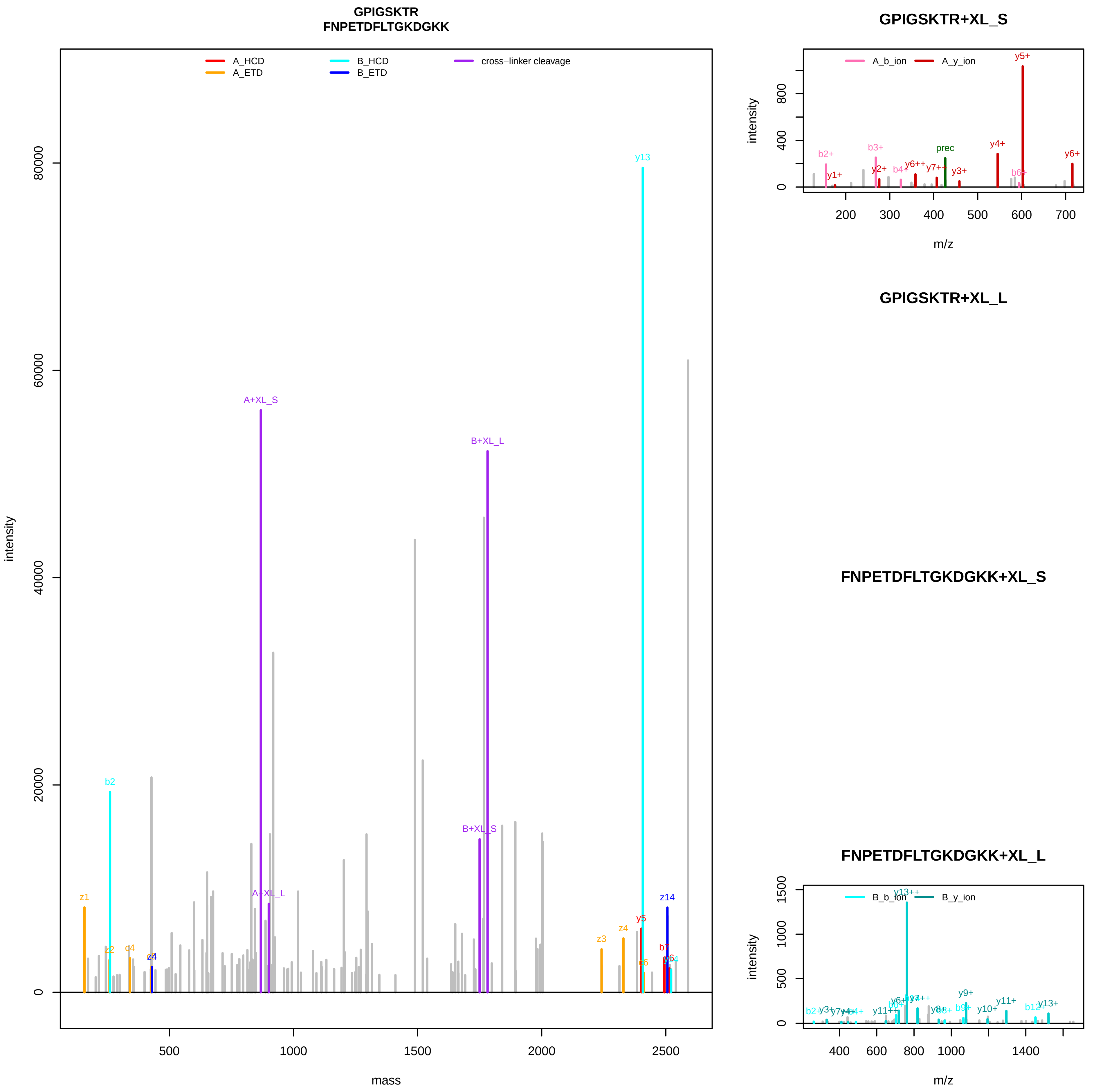

GPIGSKTR
FNPETDFLTGKDGKK
A_HCD
A_ETD
B_HCD
B_ETD
cross−linker cleavage
A+XL_S
B+XL_L
B+XL_S
A+XL_L
y13
b2
z1
z2
c4
z4
z3
z4
y5
c6
b7
y6
z14
intensity
mass
GPIGSKTR+XL_S
A_b_ion
A_y_ion
b2+
y1+
b3+
y2+
b4+
y6++
y7++
prec
y3+
y4+
y5+
b6+
y6+
m/z
GPIGSKTR+XL_L
FNPETDFLTGKDGKK+XL_S
FNPETDFLTGKDGKK+XL_L
B_b_ion
B_y_ion
y13++
y9+
y11+
y13+
y10+
b12+
y11++
m/z
